# The effects of a mindfulness-based lifestyle program for adults with Parkinson’s disease: a mixed methods, wait list controlled randomised control study

**DOI:** 10.1186/s12883-016-0685-1

**Published:** 2016-09-08

**Authors:** Jenny Advocat, Joanne Enticott, Brooke Vandenberg, Craig Hassed, Jennifer Hester, Grant Russell

**Affiliations:** 1 Southern Academic Primary Care Research Unit, School of Primary Health Care, Monash University, Bldg 1, 270 Ferntree Gully Rd, Notting Hill, Victoria 3168 Australia; 2 Department of Psychiatry, Southern Synergy, Monash University, 126-128 Cleeland Street, Dandenong, Victoria 3175 Australia; 3 School of Primary Health Care, Monash University, Bldg 1, 270 Ferntree Gully Rd, Notting Hill, Victoria 3168 Australia; 4 Department of General Practice, School of Primary Health Care, Monash University, Bldg 1, 270 Ferntree Gully Rd, Notting Hill, Victoria 3168 Australia; 5 Department of Family Medicine, University of Ottawa, Ottawa, ON Canada; 6 Brotherhood of St Laurence, Melbourne, Australia

**Keywords:** Parkinson’s disease, Neurological disorder, Mindfulness, Group program, Meditation

## Abstract

**Background:**

Parkinson’s disease (PD) is the second commonest neurodegenerative disease in developed countries. Current treatment for PD is pharmacologically focused and can have significant side-effects. There is increasing interest in holistic approaches including mindfulness to help manage the challenges associated with living with PD. We hypothesised that there would be an improvement in PD associated function and wellbeing in participants after participating in a 6-week mindfulness-based lifestyle program, and that these improvements would be sustainable at 6 months. Our primary objective was to determine changes in function and wellbeing associated with PD.

**Methods:**

An exploratory prospective, mixed-method, randomised control trial incorporating a before and after design with a waitlist control, with an embedded qualitative component was conducted in 2012–2013. Participants included community living adults with disability congruent to H&Y Stage 2 PD, aged 18–75, fluent in spoken and written English and able to attend at least four of six sessions of the program. Participants were randomised to the intervention or wait-list control groups at two locations. All participants in the wait-list control group eventually received the intervention. Two randomisation codes were created for each location. Allocation to the intervention or wait-list control was by random number generation. The program facilitator and participants were blinded to participant data.

**Results:**

Group 1 included 35 participants and group 2 (the waitlist control), 37. Data was analysed from 24 (group 1) and 33 (group 2) participants. The intervention group, compared to the waitlist control, showed a small improvement in function and wellbeing associated with PD immediately after the program (t-score = −0.59) and at 6-month post intervention (t-score = −1.42) as reported by the PDQ-39 SI. However this finding was not significant (*p* = 0.56 and 0.16 respectively). A small yet significant effect size (*β* = 0.23) in PDQ-39 ADL was reported in group 1 after 6-months post-intervention. This showed a positive improvement in the ADL as reported by group 1 after 6-months (t-score −1.8, *p* = 0.04). Four secondary measures are reported.

**Conclusions:**

Our findings suggest mindfulness-based lifestyle programs have potential to assist participants in managing the ongoing difficulties associated with a neurological condition such as Parkinson’s disease. Importantly, our study shows promise for the long term benefits of such programs. Improvements to participant activities in daily living and mindfulness were retained at 6-months post intervention. A more definitive study should be conducted in a larger sample of PD patients to further explore these findings and their impact on reducing stress and anxiety in PD patients.

**Trial Registration:**

Australian New Zealand Clinical Trials Registry (ANZCTR) ACTRN12612000440820, 17^th^ April 2012.

## Background

Parkinson’s disease (PD) is the second commonest neurodegenerative disease in developed countries. [1]. When adjusted by age, global incidence rates of PD range between 9.7 and 13.8 per 100,000 population [2]. In 2014 there were nearly 70,000 people living with PD in Australia, approximately 14,500 more than in 2005, and 5,100 more than in 2011 [3]. With many developed countries reporting increased ageing populations, the incidence of PD is expected to increase [4, 5]. Despite this recognition, many diagnosed with PD continue to suffer poor quality of life, experiencing complex motor and non-motor symptoms affecting emotional, cognitive and physical well-being [6].

Where PD is poorly self-managed, symptoms can lead to significant challenges in completing basic daily activities. Negative outcomes for people diagnosed with PD extend beyond the individual or patient level. Large proportions of the burden of disease are carried by those who care for PD sufferers and economically by health-systems [2, 7]. These effects can be intensified by complex and, at times, invasive treatment options. Lifestyle-based interventions, incorporating mindfulness training, have been identified as improving self-management outcomes in patients with a variety of chronic [8–12] and neurological diseases [13, 14].

Mindfulness is a form of meditation that has been defined as: “*the awareness that emerges through paying attention on purpose, in the present moment, and nonjudgmentally to the unfolding of experience moment by moment*” [15]. One program for multiple sclerosis, utilising lifestyle and mindfulness training in conjunction with conventional medical care, found reduced costs of disease, reversal of disease progression and improvement in patient quality of life [16]. Meta-analyses also suggest longer term improvements in patient mental health [12]. In more recent years, programs encompassing mindfulness have begun to explore the neurobehavioral effects for people living with PD, with positive outcomes, including improved motor function, patient experience of pain [17] and development of better coping mechanisms [18]. The longitudinal benefit of such programs on the self-management of PD however, to the best of our knowledge, has not yet been explored.

This study aimed to investigate the impact of a 6 week mindfulness-based lifestyle program for community living adults with Hoehn and Yahr (H&Y) stage two PD [19]. Our primary objective was to determine changes in function and wellbeing associated with PD. Secondary objectives were changes in health behaviours, mental health, and locus of control.

We hypothesised that there would be an improvement in PD associated function and wellbeing in participants after participating a 6 week mindfulness-based lifestyle program and that these improvements would be sustainable at 6 months.

## Methods

### Study design

The study was an exploratory prospective, mixed-method, randomised clinical trial incorporating before and after design with a waitlist control, with an embedded qualitative component. The current paper presents the study’s quantitative data and findings. Qualitative findings will be reported separately. The intervention was participation in a 6-week mindfulness-based lifestyle program. All participants in the wait-list control group eventually received the intervention, but only after the intervention group. The study was conducted over 12 months between 2012 and 2013. A detailed description of the methodology has been previously published [20].

### Advisory Group

An expert advisory group was formed to provide advice on recruitment and link clinicians and researchers. Apart from contributions to the Advisory Group, the funding body, Parkinson’s Victoria (PV), had no other involvement in the decisions and procedure concerning data collection, analysis, interpretation and academic dissemination.

### Setting

The intervention was delivered at two venues, located in two different inner-urban suburbs of Melbourne, Australia. Melbourne is a capital city in Australia with over four million people. In Australia, there are approximately 80,000 people living with Parkinson’s. In Victoria, more than 2,225 people are newly diagnosed with Parkinson’s every year [21]. The suburbs chosen for the study were located close to Parkinson’s Victoria meeting group sites.

### Participants

The intervention group were participants attending the first 6-week mindfulness-based lifestyle program at each location. The control participants were those on a wait-list control group at each location. All the control participants were invited to attend the same program held at each site, but only after the intervention participants had completed the program.

Eligibility criteria were:

- Between the ages of 18 and 70 at the time of recruitment
- Fluent in spoken and written English
- Able to attend at least four of six sessions of the mindfulness-based lifestyle program
- Community living adults with disability congruent H&Y Stage 2 PD [19]. This staging, for the purposes of the study, was determined by screening participants using the two below questions developed in conjunction with a neurologist. To be eligible, participants had to answer ‘yes’ to both questions:
  - ○ ‘Do you have problems of shaking (tremor), stiffness or difficulty with movements on both sides of your body?’
  - ○ ‘Most of the time, can you walk straight and stand up without assistance?’

### Recruitment

Volunteer participants were recruited from the PD community residing in metropolitan Melbourne. Target participants were initially invited to participate through a combination of written invitations from Parkinson’s Victoria sent to people listed on the Parkinson’s registry, advertisements in PV publications and through neurologists and primary care clinicians. In order to assist with the potential issue of slow recruitment, as seen in previous trials [22], a recruitment officer (BV) was employed. The recruitment officer worked with the Advisory Group to develop additional recruitment strategies, including: the development of promotional materials (flyers, brochures, and posters), engaging with PD support groups, presenting at PD community events and utilising the M.J. Fox Foundations clinical trial finder [23].

### Intervention

**Fig. 1:**
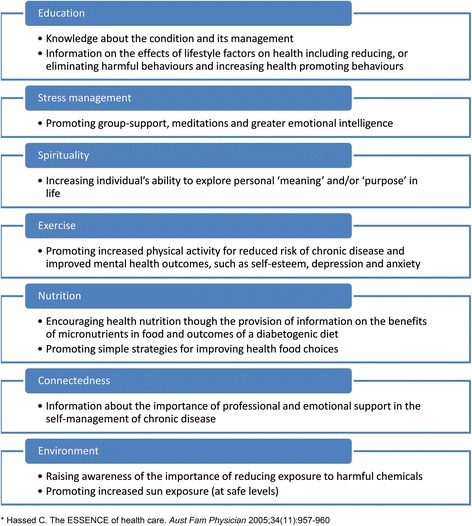
ESSENCE

The intervention was a mindfulness-based lifestyle program involving facilitated, two hour group sessions once a week for a total of 6-weeks. The program was designed to introduce key lifestyle and mindfulness elements to participants. Typically, each session consisted of a 5–20 min mindfulness practice, led by the program facilitator and author of the model (CH), introduction to one of the ESSENCE elements (Fig. 1) and an open group discussion about participants’ thoughts and feelings about the program. The mindfulness practice included specific techniques, such as, attention to the breath, a ‘body scan’ and letting go of competing or negative thoughts. Participants were further introduced to strategies designed to help them to live better with a chronic disease and encouraged to use aspects of the program that were most relevant to them.

A 3 disc CD pack with guided mindfulness practices free-of-charge and printed information was given to participants to assist them with their practice outside of the group sessions. Participants were expected to continue their usual medical management and monitoring by their general practitioners and specialists.

Each intervention session was 2 h long and attended by a research assistant (BV) in addition to the course facilitator (CH). The research assistant was responsible for completing the attendance role, collecting observational data (discussed further below) and preparing the morning or afternoon tea.

### Data collection

#### Quantitative data

The primary outcome is the change in function and well-being associated with PD, measured by the validated Parkinson’s disease Questionnaire (PDQ-39) [24], which captures information about PD-related symptoms and wellbeing. The questionnaire has 39 items each on a scale of 0 (never) to 5 (always or cannot do at all). Eight dimensions are reported: mobility (MOB) (10 items), activities of daily living (ADL) (6 items), emotional wellbeing (EMO) (6 items), stigma (STI) (4 items), social support (SOC) (3 items), cognitions (COG) (4 items), communication (COM) (3 items), and bodily discomfort (BOD) (3 items). A summary index score (PDQ-39-SI) provides an overall picture of health-related-quality-of-life (HRQoL).

Secondary outcomes are changes in health behaviours, mental health and locus of control measured by four validated, self-administered questionnaires. 1. mindfulness as measured by the Freiburg Mindfulness Inventory (FMI) [25], 2. depression, anxiety and stress as measured by the DASS 21 [26], 3. locus of control as measured using the multidimensional locus of control (LOC), Form B [27], and 4. exercise and nutrition as measured in the Health Behaviours Questionnaire (HBQ)) [28].

All participants completed the study questionnaires at three time points: (T0) baseline, (T1) week 7 after the intervention participants (but not wait-list controls) had completed the intervention and (T2) 6 months after the intervention had completed for all participants (both intervention and wait-list controls).

Study questionnaires were mailed or given to participants in person. Questionnaire packages included a self-addressed return envelope, cover letter with contact details for the researchers and a request that questionnaires be completed and returned within 1 week. Baseline (T0) questionnaires were mailed to all participants. At this time, participants were also emailed a link to an electronic instructional video, detailing each component of the questionnaire. Week 7 (T1) questionnaires were distributed to the intervention participants after the final session and if a participant was absent then the questionnaire was mailed. Wait-list control participants received the questionnaire in the mail. The 6 month (T2) questionnaires were all mailed. Follow up strategies were implemented if a survey was not returned within 2 week of the sent date, consisting of phone calls and emails to the participant made by a research assistant. Follow up was ceased at 4 weeks post sent date.

#### Other quantitative data

We monitored participant attendance at the weekly intervention sessions. If a participant did not attend four or more sessions, this was recorded and their data excluded from the analysis. We also monitored participant adherence using a modified medical outcomes study [29] and by participants self-rating their practice of the program exercises in the week prior on a 5-point Likert scale. Intervention participants were required to complete the adherence measure prior to leaving the fourth and final sessions. All participants were mailed the adherence measure along with the other study questionnaires at the 6-month post intervention point.

### Sample size

In order to detect a change in the primary outcome measure of PDQ-39 having a change degree consistent with a medium-to-large effect in this study, a sample size of 80 participants was needed. This calculation assumed a dropout rate of 25 %, which is similar to rates experienced by other studies involving participants with PD [22]. See the protocol paper for more information about the power and sample size calculations [20].

### Randomisation

Participants were randomised to either the intervention or wait-list control groups at each location a week prior to commencement of the first programme. Two randomisation codes were created for each location. Allocation to the intervention or wait-list control was by random number generation (www.randomizer.org) using the permuted four block method.

### Blinding

The programme facilitator (CH) and participants were blinded to all participant data.

### Analysis

#### Quantitative data

At the 7-week data collection time point, intervention participants had completed the 6-week intervention but the wait-list controls had not; therefore analysis at this stage followed that of a standard two group RCT. This study used the baseline and 7-week data points and paired t-tests to examine differences in the outcome measures. At the 6-month data collection point, all participants in both groups had completed the intervention; therefore we did a before and after paired data analysis using baseline and 6-month data points to examine any long-term differences in the outcome measures. Effect size for the difference between the two groups was calculated using the t-statistic from the *t*-test using the pooled variance of the variables [30].

Additionally, outcomes of the programme were correlated with participant scores for adherence using Pearson’s regression. We set a significance level of alpha = 0.05 for all outcomes in this exploratory study in order to identify any trends in the results.

### Standard protocol, approvals, registration and patient consents

The study was approved the Monash University Human Research Ethics Committee project number CF11/2662-2011001553 and registered with the Australian and New Zealand Clinical Trial Register at http://www.anzctr.org.au/, ACTRN12612000440820, 17^th^ April 2012. All participants provided written informed consent prior to study involvement.

## Results

**Fig. 2:**
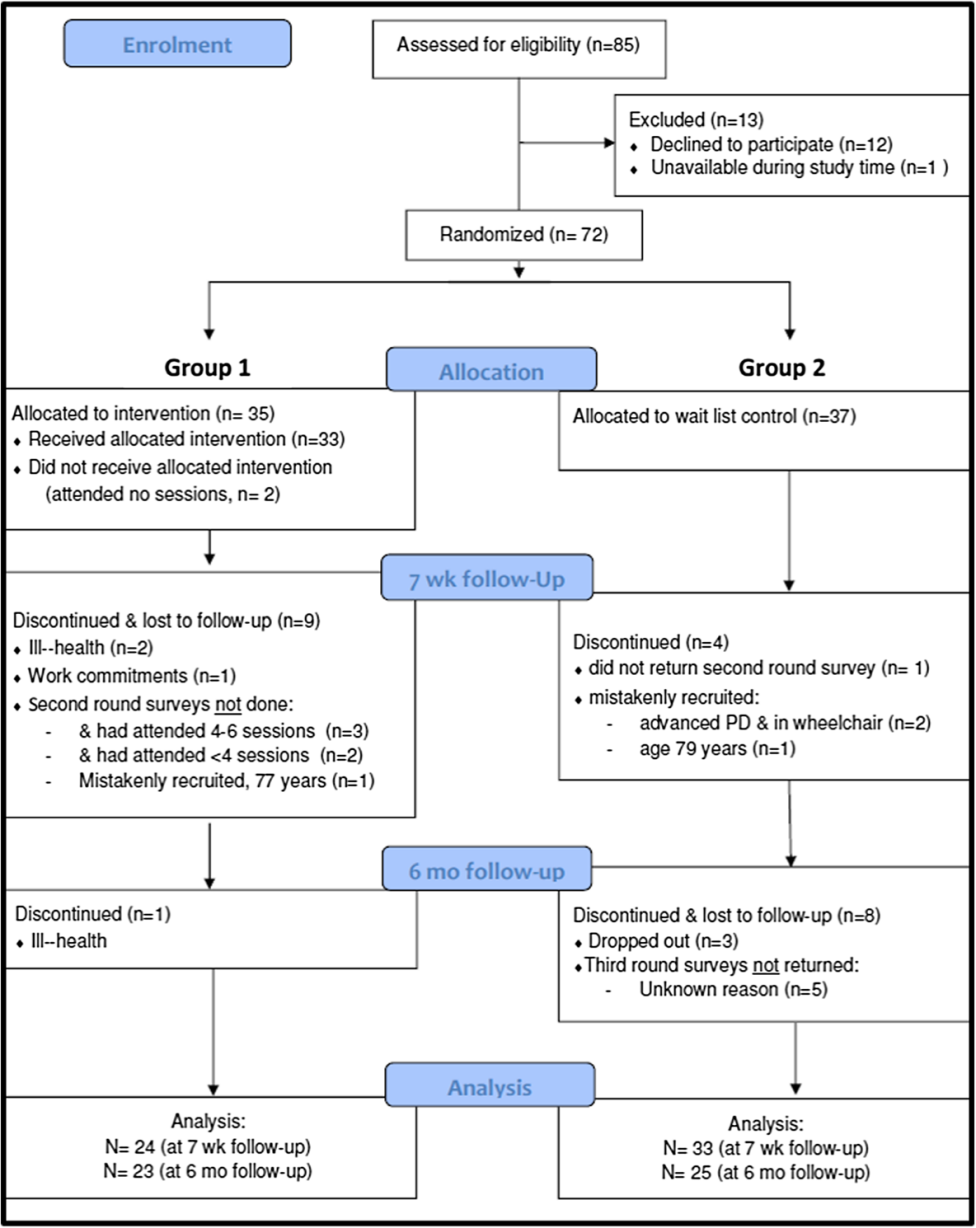
Flow of participants

Initial study eligibility criteria included an age limit for participants of 18–70 years. At the recommendation of the Advisory Group, the upper age limit was raised to 75 years to maximise recruitment and improve study power. Eighty-five participants were initially recruited. Twelve declined to participate, one was unavailable at the time of the study. Of the 72 randomised and invited to attend a session, 57 participants completed the intervention and 23 participants contributed to study data at 6-months (Fig. 2). At baseline the intervention and wait-list control groups were similar with regard to most demographic characteristics and baseline outcomes (Tables 1 and 2).

**Table 1:** Baseline characteristics of ESSENCE participants

Baseline characteristics of ESSENCE participants		Intervention group	Wait-list control group	Total (*n* = 57)
Gender	Female, % (*n*)	66.7 % (16)	51.5 % (17)	57.9 % (33)
Age	Age year, mean (sd)	62.8 % (7.6)	63.7 % (8.6)	63.3 (8.1)
	range	46–74	39–75	39–75
PD onset	Age at onset, mean (sd)	55.8 % (8.8)	55.1 % (10.5)	55.7 (9.9)
	range	37–70	33–73	33–73
Current smoker	yes, % (*n*)	0 % (0)	6.1 % (2)	3.5 % (2)
Site	Camberwell, % (*n*)	70.8 % (17)	69.7 % (23)	70.2 % (41)
	Essendon, % (*n*)	26.2 % (7)	30.3 % (10)	29.8 % (17)
COB	Born in Australia, % (*n*)	67 % (16)	79 % (26)	74 % (42)
Marital status	Married/defacto, % (*n*)	71 % (17)	79 % (26)	75 % (43)
Education	Above secondary school, % (*n*)	67 % (16)	85 % (28)	77 % (44)

**Table 2:** Main outcome summary. Group 1 received the intervention first and Group 2 was the waitlist control. Group 2 received the intervention after the 7-week measures were collected

OutcomeS		Group	Baseline level, mean (SD)	7-week change (95 % CI)	6-Month follow-up change (95 % CI)	7-week effects (change comparison between groups)			6-Month follow-up effects (change from pre-intervention all individuals)		
						t	*p* Value	Effect size	t	*p* Value	Effect size
Parkinson’s symptoms	PDQ39-SI	Intervention group	22.2 (12.4)	−0.54 (−3.41 to 2.32)	−0.89 (−3.71 to 1.93)	−0.59		-	−1.42	0.16	-
		Wait-list control	26.8 (17.5)	−1.53 (−3.64 to 0.57)	−2.54 (−6.76 to 1.67)						
	PDQ39-ADL	Intervention group	20.8 (14.6)	−2.43 (−8.11 to 3.25)	−2.54 (−6.87 to 1.80)	0.15	0.89	-	−1.80	0.04 ^b,a^	0.23^b^ (small)
		Wait-list control	26.9 (23.6)	−2.02 (−4.66 to 0.62)	−4.17(−10.75 to 2.42)						
Mindfulness	FMI	Intervention group	37.1 (8.2)	4.88 (1.95 to 7.80)^a^	0.95 (−1.91 to 3.82)	−3.02	<0.01^a^	0.61 (medium)	2.35	0.02^a^	0.32 (small)
		Wait-list control	34.5 (8.8)	−1.06 (−3.81 to 1.68)	4.74 (0.67 to 8.81)						
Depression (D) Anxiety (A) Stress (S)	DASS-D	Intervention group	4.50 (5.22)	1.92 (0.201 to 3.63)^a^	0.78 (−0.90 to 2.47)	−0.66	0.51	-	−0.62	0.54	-
		Wait-list control	7.19 (7.83)	1.06 (−0.84 to 2.97)	−1.75 (−4.63 to 1.13)						
	DASS-A	Intervention group	7.58 (4.79)	0.33 (−1.67 to 2.34)	−0.26 (−1.79 to 1.27)	−0.62	0.54	-	−0.89	0.38	-
		Wait-list control	9.81 (7.56)	−0.63 (−2.92 to 1.67)	−0.92 (−3.21 to 1.38)						
	DASS-S	Intervention group	8.78 (6.35)	2.17 (0.12 to 4.23)^a^	−1.0 (−2.89 to 0.89)	−2.61	0.01^a^	0.32 (small)	−1.80	0.04 ^b,a^	0.27^b^ (small)
		Wait-list control	12.44 (9.65)	−1.63 (−3.68 to 0.43)	−1.42 (−3.58 to 0.75)						
Locus of control: Internal (I) Chance (C) Doctors (D) Powerful Others (O)	LOC-I	Intervention group	19.6 (5.5)	0.13 (−1.79 to 2.04)	0.91 (−1.92 to 3.75)	0.99	0.33	-	0.07	0.94	-
		Wait-list control	16.5 (5.5)	1.5 (−0.50 to 3.50)	−0.78 (−3.19 to 1.62)						
	LOC-C	Intervention group	16.0 (7.2)	1.33 (−1.09 to 3.76)	1.14 (−1.83 to 4.10)	−1.69	0.10	-	0.73	0.47	-
		Wait-list control	18.6 (6.4)	−1.09 (−2.88 to 0.70)	0.39 (−2.75 to 3.53)						
	LOC-D	Intervention group	12.7 (3.7)	0.63 (−0.88 to 2.13)	0.22 (−1.11 to 1.56)	−1.80	0.04 ^b,a^	0.28^b^ (small)	0.39	0.70	-
		Wait-list control	11.8 (3.3)	−0.91 (−1.98 to 0.16)	0.16 (−1.34 to 1.66)						
	LOC-O	Intervention group	8.0 (3.7)	0.13 (−1.38 to 1.63)	0.23 (−1.30 to 1.76)	0.064	0.95	-	0.40	0.69	-
		Wait-list control	8.0 (3.0)	0.18 (−0.93 to 1.29)	0.17 (−1.21 to 1.54)						
Exercise (hours)		Intervention group	1.4 (2.0)	0.38 (−0.22 to 0.97)	−0.28 (−1.14 to 0.58)	−1.03	0.31	-	0.6717	0.50	-
		Wait-list control	1.7 (2.4)	−0.32 (−1.41 to 0.77)	0.86 (−0.54 to 2.25)						
Nutrition (fruit serves/day)		Intervention group	2.2 (1.2)	0 (−.41 to 0.41)	0.32 (−0.05 to 0.69)	0.68	0.50	-	−1.80	0.04 ^b,a^	0.28 (small)
		Wait-list control	2.4 (1.3)	0.21 (−0.24 to 0.66)	0.29 (−0.31 to 0.90)						

^a^significant finding. ^b^ one-sided finding. Effect size is the Cohen's d: small ≥ .20, medium ≥ .50, large ≥ .80

Severity of PD as measured at baseline by the PDQ39 was consistent with H&Y stage 2 PD in both intervention and wait-list control groups. Mindfulness, as indicated by FMI scores, was consistent with the normal Australian population in both groups [25] and, on average, the wait-list controls were more *mindful* than the intervention individuals as indicated by a small yet significantly better FMI average score at baseline (*t* = 2.31, *p* = 0.04). Depression and stress average scores at baseline were consistent with the Australian population [31], but average anxiety scores were poorer than the Australian population and consistent with ‘mild’ degree anxiety. Overall, 16 % (9/56) of all participants had moderate or greater depression, 16 % (9/55) had moderate stress and 39 % (22/56) had moderate anxiety. The wait-list controls on average appeared to have poorer depression, stress and anxiety scores however differences were not significant. For the four dimensions of locus of control (Internal (I), Chance, Doctors, Powerful Others), both groups had similar average scores at baseline, except for one dimension, the internal (LOC-I) aspect for which there was a slight but significant difference between the two groups (*t* = −2.06, *p* = 0.04). Both groups reported similar average hours of exercise per week and average nutritional intake of fruit serves per day (Table 2).

### 7-week impact of intervention, RCT analysis

The analysis at 7-weeks followed that of a standard two group RCT and change scores compared to baseline are shown in Table 2. There was no difference in the primary outcome of PDQ39 since baseline, nor was there a difference between the intervention and wait-list control groups.

Mindfulness significantly improved in the intervention group after completing the intervention, showing a significant difference compared to the wait-list controls (*t* = −3.02, *p* < 0.01) and a medium effect size (Cohen's *d* = 0.61). Mental health, as measured by the DASS, suggested a small increase in depression in the intervention group since baseline, although this difference was not significantly different between groups (*t* = −0.66, *p* = 0.51). Anxiety scores on average did not change. However, stress significantly increased in the intervention group after completing the intervention, showing a significant increase compared to the wait-list controls (*t* = −2.61, 0.01) and a small effect size (Cohen's *d* = 0.32).

Locus of control measures showed a significant difference between groups in one dimension only, the internal aspect (LOC-I). This represented a significant increase in perceived internal control in the intervention group after completing the intervention compared to the wait-list controls (*t* = −1.8, *p* = 0.04) and a small effect size (Cohen's *d* = 0.28). Health behaviours as measured by average weekly exercise and daily fruit intake did not change.

### 6-month impact of intervention, pre- post analysis

The 6-month analysis differed from the 7-weeks analysis because all participants had received the intervention approximately 6 months prior; therefore this analysis was a before and after examination of scores using paired t-tests and all participants. Comparing the primary outcome, PDQ39, there was no measurable difference at 6 months compared to baseline scores. Of the eight dimensions of the PDQ39, only one suggested a possible improvement since baseline had occurred on average, and this was the activities in daily life (PDQ39-ADL) sub-category (*t* = −1.8, *p* = 0.04) and a small effect size (Cohen's *d* = 0.28).

Increased mindfulness on average was measured at 6 months, and this results was significant (*t* = 2.35, *p* = 0.02) with a small effect size (Cohen's *d* = 0.32).

Decreased stress on average was measured at 6 months, and this results was significant (*t* = −1.8, *p* = 0.04) with a small effect size (Cohen's *d* = 0.28).

Health behaviours as measured by average daily fruit intake improved, and this improvement was significant (*t* = −1.8, *p* = 0.04) with a small effect size (Cohen's *d* = 0.28). The average increase in fruit intake corresponded to an extra 1/3 serves extra per day or approximately 2 serves extra per week.

### Adherence analysis

**Fig. 3:**
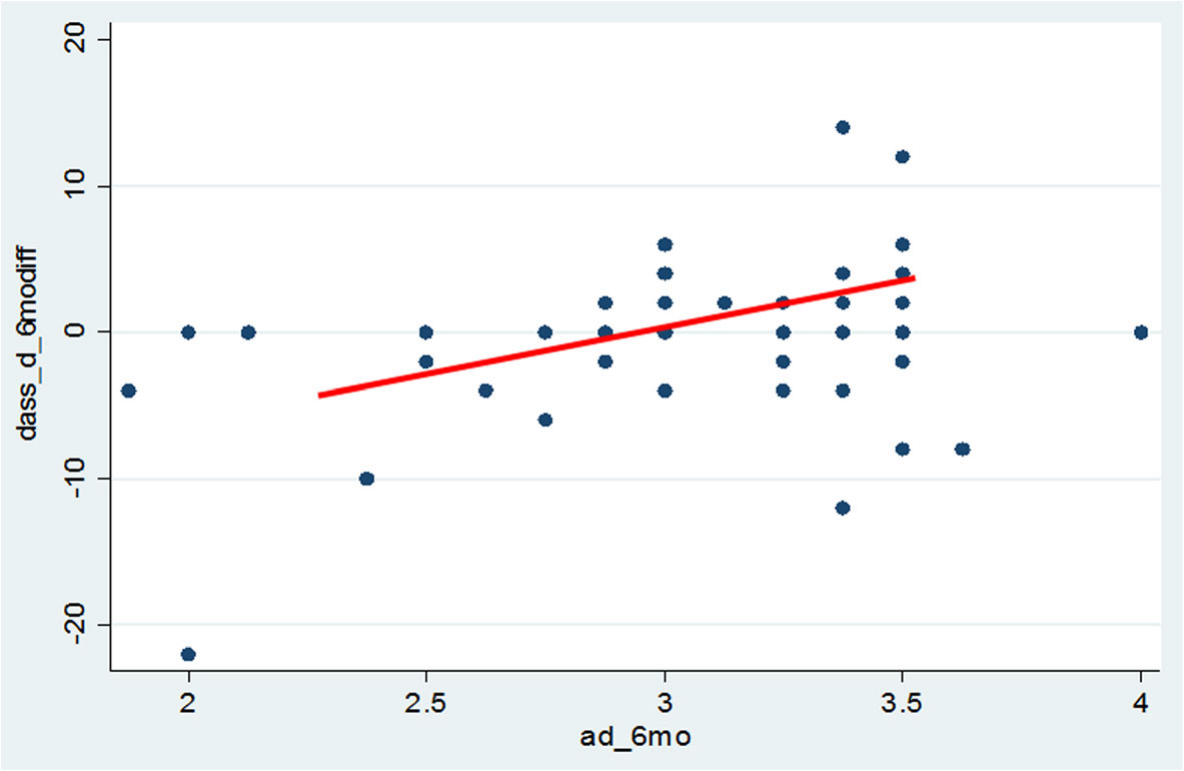
Adherence to the program (*x-axis*) and depression scores (*y-axis*) at 6 months after program completion. High adherence scores represent more practice of the program principles. Positive depression change scores represent greater depression at 6 months compared to baseline. The correlation between increased depression and adherence was weak, yet significant (Pearson’s *r* = 0.3, *p* = 0.02

Adherence data was plotted against the study measures. There were no significant correlations between adherence and any outcome measures during the program or in the week after the 6-week program. The 6 month difference scores showed no significant correlations with adherence except for the depression (DASS-d) measure, see Fig. 3. The correlation with increased depression and adherence was weak yet significant (Pearson’s *r* = 0.3, *p* = 0.02) at 6 months.

## Discussion

Involvement in a mindfulness-based lifestyle program for community living adults with H&Y [19] stage 2 PD resulted in a significant increase in mindfulness*,* stress-management and improvement in activities of daily living at 6 month follow up. This positive effect on participant motor function is not uncommon where behaviour-change is the central focus of the intervention [32]. A similar study exploring the effects of the provision of behavioural advice on PD symptom management found improved motor performance and reduced tremor amongst PD participants when compared to a control group [33]. Furthermore, improvements in function may be found where advice on behaviour-change is delivered within a supportive group setting [33, 34]. The embedded qualitative study highlighted the group support that was part of the program had assisted participants in coping with PD, particularly when groups shared chronic disease management ‘tips’ and experiences [35, 36]. The positive group dynamic of the mindfulness-based lifestyle program delivered in our study offered opportunities for participants to share ideas for better disease management and may be reflected in the significant increase in participants’ ADL after 6-months from receiving the program. This sustained effect was similarly noted in participant improvement in emotional well-being. Positive outcomes for motor and non-motor dysfunction are particularly reflected where mindfulness is strongly embedded [37] and emphasised in intervention programs. Others have found that interventions which promote personal control may assist in reducing disability and improving quality of life in people with PD [38].

Emerging evidence which suggests that mindfulness may lead to neuroplastic changes of regions in the brain involved in the regulation of emotion [39, 40] and improvements in emotional well-being, particularly stress-management, anxiety and depression [36, 41, 42] for people with chronic and neurological disease. Our study however showed a statistically but not clinically significant increase in depression following the intervention which returned to normal at 6 months. In the initial stages of learning mindfulness people can report more awareness of depressive symptoms that were already present [43]. This may be further compounded by negative effects in changing PD medication dosage [44] and the challenges associated with adapting to chronic disease, whereby making adjustments to a person’s social and work life can be stressful or create worry until the benefits of those changes start to make themselves obvious. It should be noted that severity of PD symptoms is evidenced to fluctuate throughout the year [45] with poorer outcomes reported during busy social periods, such as Christmas, which is when our study took place. There also appeared to be a correlation between increased adherence to the program and depression at 6 months, which may need to be considered as a possible unwanted outcome in a bigger study.

We also found an increase in stress immediately after the 6-week program and then significant reduction compared to baseline in stress at 6 months post program, primarily associated with a recent PD diagnosis. Importantly these benefits appeared to be sustainable over longer periods of time signifying indicating the potential to improve mental well-being in those recently diagnosed with PD. Early treatment strategies which aid in the diminishment of symptom severity, or the stress associated in experiencing symptoms, are seen to have more meaningful impacts in improving quality of life [46].

### Mindfulness: a feasible therapeutic intervention

We found that mindfulness training may be a feasible therapeutic intervention within a population of persons with PD. Previous evidence has suggested greater benefits from mindfulness training particularly for depression but only after sustained or formal practice [47]. Our findings indicate that by simply introducing mindfulness practices, benefits for people with PD can be achieved. After participating in only six sessions of a holistic lifestyle program which included up to 20 min of mindfulness training each week increases in participant mindfulness levels were found, and, importantly, appeared to be retained longitudinally. Despite many not engaging in continued formal practice, positive psychological benefits of the practice are still found [48]. For participants in this study, knowledge of mindfulness techniques assisted with better management of anxiety and stress.

Although ad hoc utilisation of mindfulness techniques (as opposed to formal practice) appeared to bring benefit to the study population, it was evident that where mindfulness was practiced more regularly and formally participants were more likely to report positive improvements to their well-being. This finding is consistent with the literature. As mindfulness skills become more integrated into a regular routine, the greater the benefit in stress reduction and symptom management [49, 50].

#### Limitations

An important limitation was the relatively small sample size of our exploratory study. A larger sample is now needed to confidently determine significant changes in patient outcomes. The programme was facilitated by the author of the model (CH) which may have introduced some bias. Furthermore, our inclusion criteria and choice of program location may have limited our study sample to only include participants of a higher socio-demographic status and lower disease severity than the general PD population and may, therefore, reduce the generalisability of our study findings. This effect is not uncommon in community based studies recruiting voluntary participants [51]. Finally, our primary outcome measure (PDQ-39) and use of the DASS-21 anxiety sub-scale may also raise some limitations. Due to the ambiguity of scoring the PDQ-39 [52, 53], and poor loading and internal consistency of the DASS-21 anxiety sub-scale, interpreting valid change magnitudes amongst a sample with early stage PD disease severity is challenging and may not reflect the true changes of disease progression from the patient perspective [54]. Larger studies should be aware of these limitation when including the PDQ-31 and DASS-21 as outcome measures.

## Conclusions

This small, community based prospective mixed-method randomised clinical trial incorporating a before and after design with a waitlist control provides needed evidence about the value of incorporating mindfulness training into the treatment options available to people with PD. Our findings suggest that mindfulness-based lifestyle programs may have great potential to assist participants in managing ongoing difficulties associated with a neurological condition such as Parkinson’s disease. In particular, our study provides suggestive evidence of potential long term benefits for the management of PD after receiving a mindfulness-based lifestyle program, when delivered alongside conventional treatment during early stages of diagnosis. A more definitive study should be conducted in a larger sample of PD patients to further explore the suggested significant increases in activities of daily living and mindfulness in reducing outcomes of stress and anxiety in PD patients.

## Abbreviations

ADL: Activities of daily living
BOD: Bodily discomfort
COG: Cognitions
COM: Communication
DASS: Depression anxiety and stress scales
EMO: Emotional well-being
FMI: Freiberg mindfulness index
H&Y: Hoehn & Yahr
HBQ: Health behaviours questionnaire
HrQOL: Health related quality of life
LOC: Locus of control
MDB: Mobility
PD: Parkinson’s disease
PDQ: Parkinson’s disease questionnaire
PV: Parkinson’s victoria
SOC: Social support
STI: Stigma
